# Microbial profiles and antimicrobial susceptibility in recurrent and non-recurrent canine otitis externa in Brazil

**DOI:** 10.14202/vetworld.2026.3976-3994

**Published:** 2026-09-11

**Authors:** Lucas Giordano Ono, Cássia Barros Pimenta, Camila Dorotea Costa, Flavia Simone Munin, Felipe Eguti de Carvalho, Ricardo Luiz Moro de Sousa, Deise Carla Almeida Leite Dellova

**Affiliations:** 1 Department of Veterinary Medicine, Faculty of Animal Science and Food Engineering, University of São Paulo, Pirassununga 13635-900, São Paulo, Brazil.; 2 Department of Animal Science, Faculty of Animal Science and Food Engineering, University of São Paulo, Pirassununga 13635-900, São Paulo, Brazil.

**Keywords:** antifungal resistance, antimicrobial resistance, Brazil, canine otitis externa, fluoroquinolones, Malassezia pachydermatis, microbial culture, Staphylococcus pseudintermedius

## Abstract

**Background and Aim:**

Canine otitis externa is a common inflammatory disorder in which bacterial and yeast proliferation may complicate treatment and contribute to recurrence. In Brazil, limited information is available comparing recurrent and non-recurrent cases while simultaneously characterizing microbial isolates and their antimicrobial susceptibility. This study compared clinical, otoscopic, and cytological findings between recurrent and non-recurrent otitis externa, identified bacterial and yeast isolates, evaluated their *in vitro* susceptibility to selected fluoroquinolones and antifungal agents, and investigated associations between clinical and microbiological variables and recurrence.

**Materials and Methods:**

Fifty-seven dogs with otitis externa presented to a veterinary hospital in Brazil were evaluated by otoscopy and cytology. Ear secretions were cultured, and isolates were identified using matrix-assisted laser desorption/ionization time-of-flight mass spectrometry. Antimicrobial susceptibility was evaluated by disk diffusion. Clinical and cytological findings were compared between recurrent and non-recurrent cases, and associations with disease status were assessed using Fisher's exact test and log-linear Poisson regression.

**Results:**

Cytology demonstrated bacterial proliferation in 54/57 (94.7%) and yeast proliferation in 25/57 (43.9%) samples. Microbial growth occurred in 46/57 (80.7%) samples, yielding 40 identified bacterial isolates and 16 *Malassezia pachydermatis* isolates. *Staphylococcus* was the predominant bacterial genus (21/40; 52.5%), and *Staphylococcus pseudintermedius* was the most frequent species (10/40; 25.0%). Resistance to both ciprofloxacin and enrofloxacin occurred in 37.5% of bacterial isolates. Among *M. pachydermatis* isolates, resistance occurred to fluconazole (43.8%), nystatin (31.3%), amphotericin B (12.5%), and ketoconazole (6.3%), whereas no isolate was resistant to miconazole. Recurrence was not significantly associated with the evaluated clinical or microbiological variables, although antifungal-resistant *M. pachydermatis* showed a nonsignificant trend toward association with recurrent disease. Cytological bacterial proliferation showed the strongest association with higher expected frequencies (RR = 18.0, p < 0.001).

**Conclusion:**

*S. pseudintermedius* and *M. pachydermatis* were the most common microorganisms recovered from canine otitis externa. The substantial fluoroquinolone resistance among bacterial isolates supports culture and susceptibility testing rather than empirical therapy. Variable antifungal resistance among *M. pachydermatis* isolates, particularly in recurrent cases, warrants further investigation. These findings provide baseline data for antimicrobial surveillance and stewardship in canine otitis externa in Brazil.

## INTRODUCTION

Canine otitis externa is a common inflammatory disorder of the external ear canal that may present as an acute or chronic condition and is highly prevalent in small animal clinical practice [1]. The condition may result from primary, predisposing, perpetuating, and secondary factors, while excessive microbial proliferation within the external ear canal can further contribute to disease chronicity [2, 3]. Treatment typically involves cleaning the external ear canal and administering topical formulations containing antibacterial, antifungal, and anti-inflammatory agents to control microbial proliferation and inflammation [4]. In some countries, including Brazil, veterinary otic products are not strictly regulated, which may contribute to their indiscriminate use. Furthermore, incomplete diagnostic investigation or inadequate treatment may favor recurrent episodes of otitis externa, resulting in repeated therapeutic interventions.

Under normal conditions, the canine external ear canal harbors commensal microorganisms, including bacteria and yeasts. However, the composition of the ear canal microbiota may change during otitis externa, resulting in the predominance of microorganisms such as *Staphylococcus* spp., *Pseudomonas aeruginosa*, and *Malassezia pachydermatis*. Although these microorganisms are among those most frequently associated with canine otitis externa worldwide, their distribution varies according to geographic region, population characteristics, and diagnostic methods [5, 6]. Identification of the predominant microorganisms in affected ear canals can facilitate the clinical management of canine otitis externa. In this context, conventional diagnostic approaches, including cytological examination of ear canal secretions combined with microbial culture and identification, may help guide treatment, particularly in recurrent cases or those showing an unsatisfactory response to therapy [3, 7]. Monitoring the antimicrobial susceptibility of isolates to antibacterial and antifungal agents commonly included in commercially available otic formulations is also important. Fluoroquinolones and azole antifungal agents are frequently incorporated into veterinary otic formulations and are therefore relevant targets for antimicrobial susceptibility surveillance in canine otitis externa.

In clinical practice, canine otitis externa may occur as a first episode or as recurrent disease. Recurrent cases are commonly associated with underlying allergic skin diseases, although other primary and perpetuating factors may also contribute [2]. According to Nuttall [3], recurrent otitis externa is defined as recurrence within 3 months after complete resolution of a previous episode. Recurrent episodes are frequently associated with bacterial and yeast overgrowth, resulting in repeated antimicrobial and/or antifungal treatment [8]. In Brazil, bacteria isolated from clinical infections, including otitis externa and pyoderma, have demonstrated multidrug resistance *in vitro* [9, 10]. Because fluoroquinolones, particularly enrofloxacin, are among the most frequently prescribed antimicrobials in veterinary medicine, resistance to this antimicrobial class has become an important focus of investigation [10]. In a recent Brazilian study, Casemiro *et al.* [11] reported that approximately 33% of *Staphylococcus* spp. isolates recovered from canine clinical infections, mainly involving the ototegumentary, urinary, and respiratory systems, exhibited *in vitro* resistance to enrofloxacin. Similar concerns have been reported for *Malassezia* spp. isolates recovered from the ear canals of dogs with otitis externa, particularly regarding their *in vitro* susceptibility to azole antifungal agents, including fluconazole, ketoconazole, and/or miconazole [12, 13].

Despite these findings, few studies have simultaneously evaluated the clinical presentation of canine otitis externa, bacterial and yeast isolates recovered using routine culture methods, and their antimicrobial susceptibility profiles to agents commonly used in veterinary otic formulations in Brazil. In particular, whether recurrent and non-recurrent cases differ in their clinical and microbiological characteristics or antimicrobial susceptibility patterns remains insufficiently characterized. Addressing this gap may provide locally relevant information for microbiological surveillance and the clinical management of canine otitis externa. Therefore, we hypothesized that recurrent canine otitis externa would be associated with distinct microbial profiles and antimicrobial susceptibility patterns compared with non-recurrent disease.

The present study aimed to compare the clinical, otoscopic, and cytological findings of dogs with recurrent and non-recurrent otitis externa; identify the predominant bacterial and yeast species isolated from the external ear canals; evaluate their *in vitro* susceptibility to selected fluoroquinolones and antifungal agents; and investigate associations between clinical and microbiological variables and disease status (recurrent versus non-recurrent).

## MATERIALS AND METHODS

### Ethical approval

All procedures involving animals were conducted in accordance with the regulations of the Brazilian National Council for the Control of Animal Experimentation and the guidelines of the Brazilian College of Animal Experimentation. The study protocol, including animal handling, clinical examinations, and sample collection, was approved by the Ethics Committee on the Use of Animals of the Faculty of Animal Science and Food Engineering, University of São Paulo, under protocol number 5223220622.

Only dogs with naturally occurring otitis externa were included; therefore, the disease was not experi-mentally induced, and no procedures that could compromise animal welfare were performed. General physical examination, otoscopic evaluation, and ear canal sampling were conducted by trained veterinarians as part of the routine diagnostic workup and caused minimal distress to the animals. The study did not involve euthanasia or invasive interventions beyond ear canal sampling. Written informed consent was obtained from each dog's owner before enrollment.

### Study period and location

Clinical examinations and ear sample collection were performed from June 2023 to December 2024 at the Veterinary Hospital of the Faculty of Animal Science and Food Engineering, University of São Paulo, Pirassununga, São Paulo State, Brazil.

### Study design

This study was an observational, cross-sectional study of dogs with naturally occurring otitis externa presented to the veterinary hospital during the study period. Dogs meeting the predefined inclusion criteria were enrolled and classified into recurrent and non-recurrent otitis externa groups according to their clinical history. All enrolled dogs underwent clinical and otoscopic examinations, followed by collection of ear canal samples for cytological and microbiological analyses. Bacterial and yeast isolates were identified, and their *in vitro* susceptibility to selected antibacterial and antifungal agents was evaluated. Clinical, cytological, microbiological, and antimicrobial susceptibility variables were subsequently compared between dogs with recurrent and non-recurrent otitis externa, and their associations with disease status were statistically assessed.

### Animals

Dogs were enrolled from the routine clinical caseload of the institution's veterinary hospital. A total of 57 dogs diagnosed with otitis externa, comprising both sexes and varying in age, breed, and body size, were included. The characteristics of the study population are presented in Table 1.

**Table 1:** Characteristics of dogs included in this study.

**Characteristics**	**Number of animals**
Total number of dogs	57
Male	29
Female	28
Breeds	
Mixed-breed	18
Shih Tzu	12
German Shepherd Dog, Labrador Retriever, and Lhasa Apso	3 for each breed
English Springer Spaniel, French Bulldog, Pomeranian, and Yorkshire Terrier	2 for each breed
Border Collie, Border Terrier, Brazilian Terrier, Golden Retriever, Jack Russell Terrier, Poodle, Pug, Siberian Husky, West Highland White Terrier, and White Swiss Shepherd Dog	1 for each breed
Age	
Up to 1 year	11
1.0–8 years	37
>8 years	9
Body weight	
Up to 10 kg	31
10–20 kg	13
>20 kg	13

Based on disease history, dogs were classified as having recurrent otitis externa (recurrence within 3 months after complete resolution of a previous episode) [3] or non-recurrent otitis externa (first episode). Of the 57 dogs, 36 (63.2%) had recurrent otitis externa and 21 (36.8%) had non-recurrent otitis externa.

The inclusion criteria were (1) clinical manifestations such as pruritus, increased local sensitivity, and/or abnormal odor in the ear canal; (2) evidence of ear canal inflammation, including erythema and swelling; (3) increased ear discharge; (4) unilateral or bilateral involvement; and (5) no topical and/or systemic treatment with antibiotics, antifungals, or anti-inflammatory drugs during the preceding 30 days to avoid interference with cytological and microbiological evaluations. Dogs with mite-induced otitis were excluded.

Dogs with digestive, respiratory, circulatory, or neurological disorders or suspected infectious diseases were also excluded.

### Otoscopic examination

After general clinical examination, the ears were carefully evaluated using an otoscope (Brasmed, Extrema, Brazil). Erythema, ear discharge, and stenosis were scored according to severity as absent (0), mild (1), moderate (2), or severe (3) [14, 15]. All evaluations were performed by the same examiner. Among dogs with recurrent otitis externa, 33/36 (91.7%) had bilateral disease, and 3/36 (8.3%) had unilateral disease. Among dogs with non-recurrent otitis externa, 18/21 (85.7%) had bilateral disease, and 3/21 (14.3%) had unilateral disease. Based on otoscopic findings, otitis externa was classified as ceruminous (31/57; 54.4%), eczematous (25/57; 43.9%), or suppurative (1/57; 1.7%).

### Sample collection

Ear samples were collected using sterile, single-use cotton swabs (Labor Import, Osasco, Brazil) inserted into the ear canal lumen and swabbed against the wall of the vertical canal. Two swabs were collected from each sampled ear: one for cytological examination and one for microbiological analysis. In dogs with unilateral otitis externa, samples for both examinations were collected from the affected ear. In dogs with bilateral otitis externa, cytological samples were collected from both ears, whereas microbiological samples were collected only from the more severely affected ear, defined by otoscopic examination as the ear with greater erythema and discharge. For dogs with bilateral disease, only cytological and microbiological findings from the more severely affected ear were included in the statistical analyses. Swab insertion was possible in all dogs, even when some degree of stenosis was present.

Additional ear canal secretion samples were collected using cotton swabs for mite detection. Each swab was rolled onto a glass slide, suspended in mineral oil, and mounted under a coverslip for microscopic examination [16]. Dogs with mite-induced otitis were excluded because the study focused on bacteria and yeasts associated with otitis externa.

### Cytological examination of ear canal secretions

Swabs collected for cytological examination were rolled onto glass slides to prepare smears, which were subsequently stained using modified Wright's stain (Quick Panoptic Kit, Newprov, Pinhais, Brazil) [17]. After staining and air-drying, the slides were examined under a light microscope (Nikon, Tokyo, Japan) at 1000× magnification. Bacterial quantity was subjectively graded using a semiquantitative scale ranging from 0 to 4 crosses per microscopic field at 1000× magnification [18]. According to the study criteria, scores of 2+, 3+, and 4+ were considered indicative of bacterial proliferation. Yeasts were counted, and >10 yeasts per microscopic field at 1000× magnification were considered indicative of proliferation [19]. Representative cytological images of bacterial and yeast proliferation in ear canal samples from dogs with otitis externa are shown in Figure 1. Five randomly selected fields were evaluated for each sample, and all cytological examinations were performed by the same examiner.

**Figure 1:**
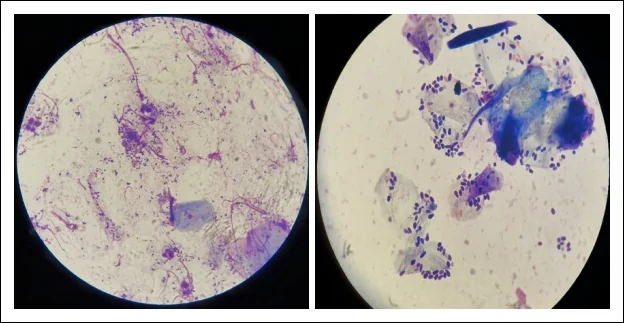
Representative cytological images showing bacterial and yeast proliferation in ear canal samples from dogs with otitis externa. Left: Grade 4+ bacterial proliferation in a 2-year-old male French Bulldog, with numerous coccoid and rod-shaped bacteria. Right: Yeast proliferation (>10 yeasts per microscopic field) in a 3-year-old male Shih Tzu. Modified Wright's stain; 1000× magnification.

### Microbiological analysis

After ear discharge collection, swabs for microbiological analysis were placed in sterile conical tubes and transported to the laboratory. Each cotton swab tip was immersed in Brain Heart Infusion (BHI) broth and subsequently used to inoculate 90-mm Petri dishes containing Nutrient agar, MacConkey agar, Mannitol Salt agar (HiMedia, Thane, India), and Sabouraud dextrose agar (Scharlab, S.L., Sentmenat, Spain) supplemented with gentamicin (100 mg/L) and chloramphenicol (100 mg/L). The plates were incubated at 37°C for 24 h for bacterial growth or 48 h for yeast growth.

### Microorganism identification

After initial incubation, bacterial colonies showing predominant growth were selected, subcultured onto Nutrient agar, and incubated at 37°C for 24 h. Yeast colonies were collected and subcultured onto Sabouraud dextrose agar supplemented with gentamicin (100 mg/L) and chloramphenicol (100 mg/L), then incubated at 37°C for 48 h. These subcultures were performed to obtain isolated colonies for subsequent microorganism identification.

All bacterial and yeast isolates were identified using matrix-assisted laser desorption/ionization time-of-flight mass spectrometry (MALDI-TOF MS) with a Bruker Microflex™ instrument (Bruker Daltonics, Bremen, Germany). Mass spectra were acquired over a range of 2,000–20,000 m/z using FlexControl 3.3 software (Bruker Daltonics). Data were processed using MALDI Biotyper 2.0 software (Bruker Daltonics) with default settings. Species- and genus-level identifications were based on comparisons of the acquired spectra with the manufacturer's reference database. Identification scores ≥2.0 were considered reliable for species level identification, whereas scores of 1.70–1.99 were considered indicative of genus level identification [20].

### Antimicrobial susceptibility testing

The susceptibility of bacteria to antibiotics and yeasts to antifungal agents was evaluated *in vitro* using the disk diffusion method according to Clinical and Laboratory Standards Institute (CLSI) guidelines, with modifications [21–23]. Two to three colonies from each culture were suspended in 0.85% sodium chloride solution to prepare an inoculum adjusted to 0.5 McFarland turbidity, corresponding to an optical density of 0.08–0.11, as determined by spectrophotometry (FEMTO SYRRUS 80MB, Sao Paulo, Brazil). The inoculum was subsequently spread evenly over the surface of Mueller–Hinton agar plates (HiMedia) in a lawn pattern. The medium was supplemented with 2% glucose and methylene blue at a concentration of 0.05 mg/mL. The plates were incubated at 37°C for 24 h to evaluate antibiotic susceptibility and 48 h to evaluate antifungal susceptibility. Results were interpreted as resistant, intermediate, or susceptible according to the diameters of the inhibition zones around the disks.

The antibiotic disks contained ciprofloxacin (5 µg) and enrofloxacin (5 µg). In addition, cefoxitin (30 µg) and oxacillin (1 µg) disks were used to determine whether *S. pseudintermedius* isolates exhibited a methicillin-resistant phenotype [24]. Zone diameter breakpoints for antibiotic susceptibility interpretation were established according to CLSI documents [22, 25] and are presented in Table 2. When CLSI breakpoints were unavailable for a given organism–antimicrobial combination, interpretive criteria were based on published literature. References supporting these criteria are provided in Supplementary Table 1.

**Table 2:** Zone diameter breakpoints for antibiotic susceptibility interpretation.

**Antimicrobial agent and bacterial category**	**Disk content**	**Resistant (mm)**	**Intermediate (mm)**	**Susceptible (mm)**
Ciprofloxacin – *Enterobacterales*	5 µg	≤21	22–25	≥26
Ciprofloxacin – Other bacteria	5 µg	≤15	16–20	≥21
Enrofloxacin – All bacteria	5 µg	≤16	17–22	≥23
Cefoxitin – *S. pseudintermedius*	30 µg	≤21	–	≥22
Oxacillin – *S. pseudintermedius*	1 µg	≤17	–	≥18

The antifungal disks contained amphotericin B (100 µg), fluconazole (25 µg), ketoconazole (50 µg), miconazole (50 µg), and nystatin (100 IU). Antifungal susceptibility was interpreted according to breakpoints defined by the supplier (Table 3), which were adapted from those established for *Candida* spp. All disks were obtained from Cefar Diagnóstica (Sao Paulo, Brazil).

**Table 3:** Zone diameter breakpoints for antifungal susceptibility interpretation.

**Antifungal agent**	**Disk content**	**Resistant (mm)**	**Intermediate (mm)**	**Susceptible (mm)**
Amphotericin B	100 µg	≤10	–	>10
Fluconazole	25 µg	≤14	15–18	≥19
Ketoconazole	50 µg	<10	10–20	>20
Miconazole	50 µg	≤11	12–19	≥20
Nystatin	100 IU	≤10	–	>10

The antimicrobial agents were selected based on the composition of the main otological products available on the Brazilian market, including ciprofloxacin and enrofloxacin as antibacterial agents and ketoconazole, miconazole, and nystatin as antifungal agents. Amphotericin B and fluconazole, routinely used in *in vitro* antifungal susceptibility assays, were included as reference compounds to compare yeast susceptibility. Quality control was performed using *Staphylococcus aureus* (American Type Culture Collection [ATCC] 23235) and *Candida albicans* (ATCC 14053).

### Statistical analysis

The characteristics of the dogs, distribution of microbial isolates according to otitis externa status (recurrent or non-recurrent), and antimicrobial susceptibility test results were described using absolute frequencies (N) and percentages (%). Age, body weight, and otoscopic and cytological scores were compared between dogs with recurrent and non-recurrent otitis externa using the Mann–Whitney U test (Wilcoxon rank-sum test). Results are presented as median (minimum–maximum) for each group, together with the corresponding p-value. A normal approximation was applied because of the presence of tied scores.

Agreement between cytological findings and culture results, as well as between ciprofloxacin and enrofloxacin susceptibility results, was assessed using Cohen's kappa coefficient. The association between otitis externa status (recurrent or non-recurrent) and each categorical variable was evaluated using Fisher's exact test. For each variable, we calculated the odds ratio (OR) for recurrent otitis externa and its corresponding 95% confidence interval (CI).

A log-linear (Poisson) regression model was used as an exploratory multivariable approach to evaluate associations between clinical and microbiological variables and the distribution of observations [7]. For each predictor, the model estimated the regression coefficient (β), standard error (SE), Z-value, p-value, and rate ratio (RR = exp β). The reference categories were male for sex, purebred for breed, and "No" for binary variables (recurrent otitis externa, bacterial proliferation, yeast proliferation, positive bacterial culture, positive yeast culture, fluoroquinolone resistance, and yeast resistance to more than one antifungal agent). Fluoroquinolone resistance and multidrug antifungal resistance were excluded from the multivariable model because these data were not available for all samples. All statistical analyses were performed using R version 4.3.3. Statistical significance was set at α = 0.05.

## RESULTS

### Characteristics of dogs with recurrent and non-recurrent otitis externa

The recurrent otitis externa group (N = 36) comprised 20 (55.6%) males and 16 (44.4%) females, including 27 (75.0%) purebred and 9 (25.0%) mixed-breed dogs. The median age and body weight were 4.0 (0.8–13.0) years and 10.7 (2.4–51.0) kg, respectively. The most common breed was Shih Tzu (N = 9; 25.0%), followed by German Shepherd Dog (N = 3; 8.3%) and Labrador Retriever (N = 3; 8.3%).

The non-recurrent otitis externa group (N = 21) comprised 9 (42.9%) males and 12 (57.1%) females, including 12 (57.1%) purebred and 9 (42.9%) mixed-breed dogs. The median age and body weight were 5.0 (0.3–13.0) years and 7.6 (2.9–44.0) kg, respectively. The most common breed was Shih Tzu (N = 4; 19.0%), followed by Lhasa Apso (N = 2; 9.5%) and Pomeranian (N = 2; 9.5%). According to the Mann–Whitney U test, median age did not differ significantly between the groups (p = 0.738), whereas median body weight was significantly higher in dogs with recurrent otitis externa (p = 0.047).

### Comparison of otoscopic and cytological scores according to otitis externa status

Median otoscopic scores for erythema, ear discharge, and stenosis did not differ significantly between dogs with recurrent and non-recurrent otitis externa (p > 0.05) (Table 4). In both groups, most dogs had mild ceruminous ear discharge and moderate erythema, whereas ear canal stenosis was uncommon. Bilateral purulent ear discharge was observed in one 2-year-old male French Bulldog with recurrent otitis externa.

Similarly, no significant differences were observed in median cytological scores for bacterial or yeast proliferation between the groups (Table 4). Cytological examination of ear secretions revealed bacterial proliferation, comprising coccoid and rod-shaped bacteria, in 34/36 (94.4%) samples from dogs with recurrent otitis externa and 20/21 (95.2%) samples from dogs with non-recurrent otitis externa. Yeast proliferation was detected in 17/36 (47.2%) and 8/21 (38.1%) samples, respectively.

### Microbiological culture and identification

Culture of 57 ear secretion samples yielded microbial growth in 46/57 (80.7%) samples. Among the culture positive samples (N = 46), 30 showed bacterial growth only, 11 showed yeast growth only, and 5 showed both bacterial and yeast growth. A total of 43 bacterial isolates were recovered from the 35 samples with bacterial growth. Of these, 40 were identified by MALDI-TOF MS, including 37 at the species level and 3 at the genus level only (*Bacillus* spp., *Pantoea* spp., and *Staphylococcus* spp.). The remaining three isolates could not be identified using this method. All 16 yeast isolates recovered from ear secretion samples were identified as *M. pachydermatis*. The origin of each ear sample and the corresponding microbiological findings are presented in Supplementary Table 2.

**Table 4:** Comparison of otoscopic and cytological scores between recurrent and non-recurrent otitis externa cases.

**Parameter**	**Recurrent otitis** **externa (N = 36)**	**Non-recurrent otitis** **externa (N = 21)**	**p-value**
Erythema (otoscopy)	2 (0–3)	2 (0–3)	0.146
Ear discharge (otoscopy)	1 (1–3)	1 (1–2)	0.636
Stenosis (otoscopy)	0 (0–3)	0 (0–2)	0.764
Bacterial proliferation (cytology)	2 (1–4)	2 (1–3)	0.549
Yeast proliferation (cytology)	0 (0–1)	1 (0–1)	0.717

N = Number of dogs or samples analyzed, as appropriate. Values are presented as median (minimum–maximum). p-values were obtained using the Mann–Whitney U test (Wilcoxon rank-sum test with normal approximation). Otoscopic scores = 0–3; bacterial proliferation scores = 0–4; yeast proliferation scores = 0 (no) or 1 (yes; >10 yeasts per microscopic field at 1000× magnification).

Table 5 summarizes the 40 identified bacterial isolates and their distribution according to otitis externa status. Overall, *Staphylococcus* was the predominant genus, accounting for 21/40 (52.5%) isolates, followed by *Bacillus* (11/40; 27.5%). The most frequently identified species were *S. pseudintermedius* (10/40; 25.0%), *Staphylococcus hominis* (5/40; 12.5%), *Bacillus licheniformis* (4/40; 10.0%), *Bacillus cereus* (2/40; 5.0%), and *Bacillus pumilus* (2/40; 5.0%). Of the 40 identified bacterial isolates, 29, representing 9 genera and 18 species, were recovered from 22 culture positive samples from dogs with recurrent otitis externa. The remaining 11 isolates, representing the genera *Staphylococcus* and *Bacillus* and comprising eight species, were recovered from 10 culture positive samples from dogs with non-recurrent otitis externa. Similarly, 11 and 5 *M. pachydermatis* isolates were recovered from the recurrent and non-recurrent otitis externa groups, respectively (Supplementary Tables 2 and 3).

Consistent with the overall distribution, *S. pseudintermedius* was the most frequently identified bacterial species in samples from dogs with recurrent and non-recurrent otitis externa, with seven and three isolates, respectively, followed by *S. hominis*, with three and two isolates, respectively (Table 5). The remaining bacterial species were identified less frequently. Among *Staphylococcus* isolates, *Staphylococcus haemolyticus* (N = 1), *Staphylococcus intermedius* (N = 1), and *Staphylococcus schleiferi* (N = 1) were identified in recurrent otitis externa samples, whereas *Staphylococcus sciuri* (N = 1) and *Staphylococcus warneri* (N = 1) were identified in non-recurrent otitis externa samples. Within *Bacillus*, *B. licheniformis* was identified in both recurrent and non-recurrent otitis externa samples (three and one isolates, respectively), as was *B. pumilus* (one isolate in each group), whereas *Bacillus megaterium* (N = 1) and *Bacillus thermoamylovorans* (N = 1), together with *B. cereus* (N = 2), were detected in recurrent otitis externa samples.

The remaining bacterial taxa detected in recurrent otitis externa samples were *Deinococcus* *proteolyticus* (N = 1), *Enterobacter cloacae* (N = 1), *Enterococcus faecium* (N = 1), *Methylobacterium* *radiotolerans* (N = 1), *Paenibacillus* *illinoisenis* (N = 1), *Paenibacillus* *provencensis* (N = 1), *Pantoea* spp. (N = 1), and *Serratia liquefaciens* (N = 1) (Table 5).

**Table 5:** Bacterial isolates identified in this study: Overall distribution and distribution according to otitis externa status.

**Bacterial identification (genus/species level)**	**Overall, No. (%)**	**Recurrent, No. (%)**	**Non-recurrent, No. (%)**
*Bacillus cereus*	2 (5.0)	2 (6.89)	0
*Bacillus licheniformis*	4 (10.0)	3 (10.34)	1 (9.09)
*Bacillus megaterium*	1 (2.5)	1 (3.45)	0
*Bacillus pumilus*	2 (5.0)	1 (3.45)	1 (9.09)
*Bacillus* spp.	1 (2.5)	0	1 (9.09)
*Bacillus * *thermoamylovorans*	1 (2.5)	1 (3.45)	0
*Deinococcus* *proteolyticus*	1 (2.5)	1 (3.45)	0
*Enterobacter cloacae*	1 (2.5)	1 (3.45)	0
*Enterococcus faecium*	1 (2.5)	1 (3.45)	0
*Methylobacterium* *radiotolerans*	1 (2.5)	1 (3.45)	0
*Paenibacillus* *illinoisenis*	1 (2.5)	1 (3.45)	0
*Paenibacillus* *provencensis*	1 (2.5)	1 (3.45)	0
*Pantoea* spp.	1 (2.5)	1 (3.45)	0
*Serratia * *liquefaciens*	1 (2.5)	1 (3.45)	0
*Staphylococcus * *haemolyticus*	1 (2.5)	1 (3.45)	0
*Staphylococcus hominis*	5 (12.5)	3 (10.34)	2 (18.18)
*Staphylococcus intermedius*	1 (2.5)	1 (3.45)	0
*Staphylococcus * *pseudintermedius*	10 (25.0)	7 (24.14)	3 (27.27)
*Staphylococcus * *schleiferi*	1 (2.5)	1 (3.45)	0
*Staphylococcus* spp.	1 (2.5)	0	1 (9.09)
*Staphylococcus * *sciuri*	1 (2.5)	0	1 (9.09)
*Staphylococcus * *warneri*	1 (2.5)	0	1 (9.09)
Total	40 (100)	29 (100)	11 (100)

Percentages in the overall column were calculated based on the total number of identified bacterial isolates (N = 40). Percentages in the recurrent and non-recurrent otitis externa columns were calculated based on the total number of isolates recovered within each group (N = 29 and N = 11, respectively).

### Agreement between cytological findings and culture results

Cohen's kappa analysis showed no agreement between cytological detection of microbial proliferation and culture positivity for either bacteria or yeasts. The kappa coefficient was −0.025 (SE = 0.064; 95% CI = −0.151–0.101) for bacteria and 0.030 (SE = 0.117; 95% CI = −0.199–0.258) for yeasts.

### Fluoroquinolone susceptibility of bacterial isolates

The ciprofloxacin and enrofloxacin susceptibility results are presented in Table 6. Of the 40 bacterial isolates, 24 (60.0%) were susceptible, 15 (37.5%) were resistant, and 1 (2.5%) showed intermediate susceptibility to ciprofloxacin. For enrofloxacin, 23 (57.5%) isolates were susceptible, 15 (37.5%) were resistant, and 2 (5.0%) showed intermediate susceptibility. All isolates resistant to ciprofloxacin were also resistant to enrofloxacin. Susceptibility classifications for the two fluoroquinolones showed complete agreement, with a Cohen's kappa coefficient of 1.00.

**Table 6:** Ciprofloxacin and enrofloxacin susceptibility of bacterial isolates.

**Bacterial identification ** **(genus/species level)**	**Ciprofloxacin ** **S**	**Ciprofloxacin ** **I**	**Ciprofloxacin ** **R**	**Enrofloxacin ** **S**	**Enrofloxacin ** **I**	**Enrofloxacin R**
*Bacillus cereus*	2	0	0	2	0	0
*Bacillus licheniformis*	3	0	1	3	0	1
*Bacillus megaterium*	1	0	0	1	0	0
*Bacillus pumilus*	2	0	0	1	1	0
*Bacillus* spp.	1	0	0	1	0	0
*Bacillus * *thermoamylovorans*	1	0	0	1	0	0
*Deinococcus* *proteolyticus*	0	0	1	0	0	1
*Enterobacter cloacae*	0	1	0	0	1	0
*Enterococcus faecium*	0	0	1	0	0	1
*Methylobacterium* *radiotolerans*	0	0	1	0	0	1
*Paenibacillus* *illinoisenis*	1	0	0	1	0	0
*Paenibacillus* *provencensis*	1	0	0	1	0	0
*Pantoea* spp.	1	0	0	1	0	0
*Serratia * *liquefaciens*	0	0	1	0	0	1
*Staphylococcus * *haemolyticus*	1	0	0	1	0	0
*Staphylococcus hominis*	0	0	5	0	0	5
*Staphylococcus intermedius*	1	0	0	1	0	0
*Staphylococcus * *pseudintermedius*	7	0	3	7	0	3
*Staphylococcus * *schleiferi*	1	0	0	1	0	0
*Staphylococcus* spp.	0	0	1	0	0	1
*Staphylococcus * *sciuri*	0	0	1	0	0	1
*Staphylococcus * *warneri*	1	0	0	1	0	0
Total	24	1	15	23	2	15

S = Susceptible; I = Intermediate; R = Resistant.

Among the 15 fluoroquinolone-resistant isolates, 10 were recovered from dogs with recurrent otitis externa: *S. hominis* (N = 3), *S. pseudintermedius* (N = 2), *B. licheniformis* (N = 1), *D. proteolyticus* (N = 1), *E. faecium* (N = 1), *M. radiotolerans* (N = 1), and *S. liquefaciens* (N = 1). The remaining five resistant isolates were recovered from dogs with non-recurrent otitis externa and comprised *S. hominis* (N = 2), *S. pseudintermedius* (N = 1), *Staphylococcus* spp. (N = 1), and *S. sciuri* (N = 1). The isolates showing intermediate susceptibility were *B. pumilus* (N = 1; enrofloxacin only) and *E. cloacae* (N = 1; ciprofloxacin and enrofloxacin), both recovered from dogs with recurrent otitis externa.

All three fluoroquinolone-resistant *S. pseudintermedius* isolates were susceptible to cefoxitin and oxacillin and therefore did not exhibit a methicillin-resistant phenotype.

### Antifungal susceptibility of *M. pachydermatis* isolates

Resistance to fluconazole (7/16; 43.8%), nystatin (5/16; 31.3%), amphotericin B (2/16; 12.5%), and ketoconazole (1/16; 6.3%) was observed among the *M. pachydermatis* isolates tested (Figure 2). No isolates were resistant to miconazole. One isolate showed intermediate susceptibility to fluconazole, whereas another showed intermediate susceptibility to both ketoconazole and miconazole. Individual antifungal susceptibility results for the 16 *M. pachydermatis* isolates are presented in Supplementary Table 3.

**Figure 2:**
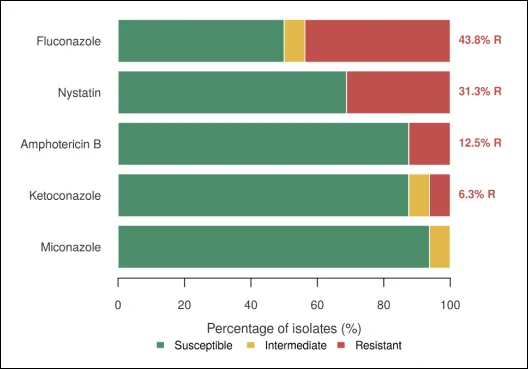
Percentage of *M. pachydermatis* isolates classified as susceptible, intermediate, or resistant to fluconazole, nystatin, amphotericin B, ketoconazole, and miconazole.

Of the five isolates recovered from dogs with non-recurrent otitis externa, one was resistant to nystatin and another showed intermediate susceptibility to fluconazole. Among the 11 isolates recovered from dogs with recurrent otitis externa, three were susceptible to all antifungal agents tested, five were resistant to one antifungal agent (four to fluconazole and one to nystatin), one was resistant to two agents (fluconazole and nystatin), one was resistant to three agents (fluconazole, nystatin, and amphotericin B) and showed intermediate susceptibility to ketoconazole and miconazole, and one was resistant to four agents (fluconazole, nystatin, amphotericin B, and ketoconazole) (Supplementary Table 3).

### Association between otitis externa status and evaluated parameters

No significant association was observed between otitis externa status and sex, breed, bacterial or yeast proliferation on cytological examination, positive bacterial or yeast culture, or the presence of fluoroquinolone-resistant bacterial isolates (p > 0.05). The association between recurrent otitis externa and the presence of *M. pachydermatis* isolates resistant to at least one antifungal agent was not statistically significant (OR = 10.67; p = 0.106) (Table 7).

**Table 7:** Association between otitis externa status and categorical variables.

**Variable**	**Recurrent**	**Non-recurrent**	**OR**	**95% CI**	**p-value**
Sex (male/female)	20 (56%)/16 (44%)	9 (43%)/12 (57%)	1.65	0.49–5.70	0.417
Breed (purebred/mixed)	27 (75%)/9 (25%)	12 (57%)/9 (43%)	2.22	0.61–8.22	0.238
Bacterial proliferation, cytology (yes/no)	34 (94%)/2 (6%)	20 (95%)/1 (5%)	0.85	0.01–17.35	1.000
Yeast proliferation, cytology (yes/no)	17 (47%)/19 (53%)	8 (38%)/13 (62%)	1.44	0.43–5.10	0.586
Bacterial culture positive (yes/no)	22 (61%)/14 (39%)	10 (48%)/11 (52%)	1.71	0.51–5.89	0.410
Yeast culture positive (yes/no)	11 (31%)/25 (69%)	5 (24%)/16 (76%)	1.40	0.42–6.90	0.762
Fluoroquinolone-resistant bacteriaᵃ (yes/no)	10 (46%)/12 (54%)	5 (45%)/6 (55%)	1.00	0.24–4.03	1.000
Yeast resistant to ≥1 antifungalᵇ (yes/no)	8 (73%)/3 (27%)	1 (20%)/4 (80%)	10.67	0.80–143.0	0.106

OR = Odds ratio for membership in the recurrent group; CI = Confidence interval. Counts represent the number of dogs or samples, as appropriate, with corresponding percentages within each group. p-values were obtained using Fisher's exact test. The unit of analysis was the dog or sample, with one sample per dog. ᵃDenominator was restricted to culture positive samples with fluoroquinolone susceptibility results (recurrent, N = 22; non-recurrent, N = 11); a sample was classified as resistant when any isolate was resistant to ciprofloxacin and/or enrofloxacin. ᵇDenominator was restricted to samples containing *M. pachydermatis* isolates with available antifungal susceptibility results (recurrent, N = 11; non-recurrent, N = 5); resistance was assessed for amphotericin B, fluconazole, ketoconazole, miconazole, and/or nystatin.

### Log-linear Poisson analysis of categorical variables related to canine otitis externa

The log-linear Poisson model identified bacterial proliferation detected by cytology as the variable with the strongest positive association with the distribution of observations (RR = 18.00; p < 0.001). Mixed-breed dogs (RR = 0.46; p = 0.007) and yeast culture positivity (RR = 0.39; p = 0.001) were significantly associated with lower expected frequencies. Recurrence had an RR of 1.71 (p = 0.050) (Table 8). Positive bacterial culture was associated with an RR of 1.59, although the association was not statistically significant (p = 0.088). No significant associations were observed for sex or cytological yeast proliferation.

**Table 8:** Log-linear Poisson regression model for canine otitis externa: Estimates, standard errors, Z-values, and p-values.

**Predictor**	**Estimate**	**95% CI**	**SE**	**Z**	**p-value**	**RR (exp β)**
Intercept	−2.814	−4.11–−1.52	0.661	−4.26	<0.001	0.06
Recurrence (yes–no)	0.539	0.00–1.08	0.275	1.96	0.050	1.71
Sex (female–male)	−0.035	−0.55–0.48	0.265	−0.13	0.895	0.97
Breed (mixed–purebred)	−0.773	−1.33–−0.21	0.285	−2.71	0.007	0.46
Bacterial proliferation (yes–no)	2.890	1.73–4.05	0.593	4.87	<0.001	18.00
Yeast proliferation (yes–no)	−0.247	−0.77–0.28	0.267	−0.93	0.355	0.78
Bacterial culture positive (yes–no)	0.464	−0.07–1.00	0.272	1.71	0.088	1.59
Yeast culture positive (yes–no)	−0.941	−1.52–−0.36	0.295	−3.19	0.001	0.39

CI = Confidence interval; SE = Standard error; RR = Rate ratio. The Poisson log-linear model was fitted to cross-classified cell counts for all categorical predictors. RR (exp β) represents the ratio of expected cell frequencies. Reference categories = Male for sex, purebred for breed, and no for all yes/no predictors. Residual deviance = 94.54; null deviance = 177.18; Akaike information criterion = 186.71; McFadden pseudo-R² = 0.466.

## DISCUSSION

### Demographic characteristics and recurrence of otitis externa

In this study, dogs with otitis externa presented during routine clinical care at a veterinary hospital in Brazil were evaluated. Most enrolled dogs were purebred and had a history of recurrent otitis externa. Consistent with the study population distribution, the log-linear Poisson model showed that mixed-breed dogs had lower expected frequencies than purebred dogs. Recurrence was associated with an RR of 1.71 (p = 0.050), reflecting the relatively high frequency of recurrent cases in the evaluated population. However, these findings represent the demographic and clinical characteristics of the study population and should not be interpreted as evidence that breed is a risk factor for otitis externa or that any evaluated variable determines recurrence because the study included only affected dogs and lacked a control population.

Previous studies have reported associations between certain breeds and canine otitis externa, potentially related to anatomical characteristics and predisposition to allergic skin diseases. Surveys conducted in the United Kingdom [1] and Germany [26] identified Golden Retrievers as a breed at increased risk of otitis externa. This predisposition may be related to anatomical characteristics, such as pendulous pinnae, which favor heat and moisture retention within the ear canal. In addition, studies conducted in Germany [27] and Korea [28] reported an increased risk of allergic skin diseases in French Bulldogs, German Shepherd Dogs, Golden Retrievers, and Shih Tzus, which may contribute to chronic inflammatory changes and microbial proliferation in the ear canals of affected dogs. These breeds were represented in the present study; however, larger epidemiological studies involving broader canine populations in Brazil are required to determine whether breed predisposition patterns reported in other countries are also present in the local canine population.

### Clinical and microbiological characteristics of recurrent otitis externa

Recurrent canine otitis externa remains an important challenge in veterinary practice because of associated complications, including pain and impaired animal welfare, chronic inflammation resulting in progressive stenosis and occlusion of the ear canal, and alterations in microbial profiles and antimicrobial susceptibility [2, 3, 7, 8]. Despite its clinical importance, recurrence was not associated with significant differences in most of the clinical or microbiological variables evaluated in the present study. Specifically, median otoscopic scores for erythema, ear discharge, and stenosis and cytological scores for bacterial and yeast proliferation did not differ significantly between dogs with recurrent and non-recurrent otitis externa. Similarly, recurrence status was not significantly associated with sex, breed, cytological evidence of bacterial or yeast proliferation, positive bacterial or yeast cultures, or the presence of fluoroquinolone-resistant bacterial isolates.

The association between recurrent otitis externa and the presence of *M. pachydermatis* isolates resistant to at least one antifungal agent was not statistically significant (OR = 10.67; p = 0.106), although the numerical difference between groups warrants further investigation. Overall, recurrence was not associated with greater clinical severity or major differences in the microbiological characteristics evaluated at the time of assessment. Given the multifactorial nature of canine otitis externa and the relatively small sample size, some associations may not have been detected because of limited statistical power. Larger longitudinal studies incorporating information on underlying diseases, previous treatments, management practices, and long-term follow-up are required to better characterize factors associated with recurrent otitis externa.

### Cytological findings and microbiological culture

Cytological examination predominantly revealed bacterial proliferation, although yeast proliferation was also detected in ear samples from dogs with both recurrent and non-recurrent otitis externa. The log-linear Poisson model identified bacterial proliferation detected by cytology as the variable with the strongest positive association with the distribution of observations (RR = 18.0; p < 0.001), consistent with the high frequency of bacterial proliferation observed cytologically in the evaluated samples. These findings agree with previous reports indicating that bacterial proliferation is a frequent cytological finding in dogs with otitis externa [29, 30].

However, agreement between cytological evidence of microbial proliferation and culture positivity was not observed for bacteria or yeasts. Although cytological examination frequently detected bacterial proliferation, bacterial culture yielded growth from 35 ear samples, and 40 of the recovered bacterial isolates were successfully identified. Differences between cytological and culture findings may partly reflect the methodologies used, as routine aerobic culture and the selection of predominantly growing colonies may have limited recovery of some bacteria from ear samples. Nevertheless, microbiological culture enabled species identification and subsequent assessment of antimicrobial susceptibility, demonstrating the complementary roles of cytological and microbiological examinations in characterizing microorganisms associated with canine otitis externa.

### Distribution of bacterial isolates

*Staphylococcus* was the predominant bacterial genus isolated from ear samples, accounting for 21/40 (52.5%) isolates, followed by *Bacillus* (11/40; 27.5%). Among the identified species, *S. pseudintermedius* was the most frequent (10/40; 25.0%) and predominated in samples from dogs with both recurrent and non-recurrent otitis externa, followed by *S. hominis* (5/40; 12.5%). Within *Bacillus*, *B. licheniformis* was the most frequently identified species (4/40; 10.0%) and was recovered from both recurrent and non-recurrent otitis externa samples.

These findings are consistent with those of another Brazilian study conducted at a different veterinary hospital, in which 782 samples collected from the canine ototegumentary system, including cases of otitis and pyoderma, were evaluated between 2019 and 2023 [11]. In that study, *Staphylococcus* spp. accounted for approximately 50% of all bacterial isolates, whereas *Bacillus* spp., *Enterococcus* spp., *Enterobacter* spp., and *Serratia* spp. together accounted for approximately 10%. These genera were also identified in the present study.

Lynch and Helbig [31] reported that isolation frequencies of *S. pseudintermedius* from canine ear samples ranged from 20% to 93.4%. In the present study, *S. pseudintermedius* accounted for 25.0% of the identified bacterial isolates. Other Brazilian studies have also identified *S. pseudintermedius* as a predominant bacterial species isolated from dogs with pyoderma and otitis, with reported frequencies ranging from 40% to 78% [9, 10, 32]. Differences in reported frequencies may partly reflect differences in study populations, objectives, and bacterial identification methods, including matrix-assisted laser desorption/ionization time-of-flight mass spectrometry or biochemical testing combined with polymerase chain reaction for species confirmation.

Studies from other Latin American countries have similarly reported frequent isolation of *Staphylococcus* spp. from canine otitis externa samples. Studies in Argentina and Paraguay reported frequencies of 36% and 45%, respectively [33, 34], whereas a retrospective study from Colombia reported *S. pseudintermedius* in 60% of ear samples [35]. However, these studies did not provide detailed descriptions of their bacterial culture and identification procedures, limiting direct comparison with the present findings.

### Predominant microorganisms associated with canine otitis externa

The present findings are consistent with previous reports from Latin America and Europe showing that *S. pseudintermedius* and *M. pachydermatis* are among the microorganisms frequently isolated from ear samples of dogs with otitis externa [33, 36–38]. Although both microorganisms can also be recovered from the ears of clinically healthy dogs, previous studies have reported higher isolation frequencies in dogs with otitis externa.

Popa *et al.* [39] reported isolation of *S. pseudintermedius* from ear samples of 40.0% of dogs with otitis externa compared with 21.1% of healthy dogs, whereas Karakaya *et al.* [6] detected this microorganism in 30.5% of dogs with otitis externa and 24.5% of healthy dogs. Similarly, Girão *et al.* [40] reported that *M. pachydermatis* was isolated more frequently from ear samples of dogs with otitis externa (57.53%) than from those of healthy dogs (30.0%). These comparisons support the frequent involvement of both microorganisms in canine otitis externa while also recognizing that they may occur in the normal ear microbiota.

### Microbial distribution according to recurrence status

Comparison of bacterial species according to otitis externa recurrence status showed a greater variety of bacterial species in ear samples from dogs with recurrent otitis externa. The most frequently isolated species in this group were *S. pseudintermedius* (24.14%), *S. hominis* (10.34%), *B. licheniformis* (10.34%), and *B. cereus* (6.89%). In dogs with non-recurrent otitis externa, *S. pseudintermedius* (27.27%) and *S. hominis* (18.18%) were the most frequently isolated bacterial species. Similarly, most *M. pachydermatis* isolates were recovered from dogs with recurrent otitis externa. However, these differences likely reflect the greater number of samples obtained from dogs with recurrent disease rather than an association between microbial isolation patterns and recurrence because positive bacterial and yeast cultures were not significantly associated with recurrence status. Future studies with larger sample sizes and culture-independent molecular approaches, such as 16S rRNA gene sequencing and metagenomic sequencing, may provide a more comprehensive characterization of microbial profiles associated with recurrent and non-recurrent otitis externa in the local canine population.

Saraiva *et al.* [7] previously evaluated the distribution of bacterial species according to otitis externa recurrence status using routine microbiological techniques. Similarly, *S. pseudintermedius* was among the predominant bacterial species recovered from dogs with both recurrent and non-recurrent otitis externa (45% and 35%, respectively). However, *Pseudomonas aeruginosa* was more frequently isolated from dogs with recurrent otitis externa (25%). Interestingly, *P. aeruginosa* was not recovered from any ear sample in the present study. This finding differs from previous reports [7, 33, 34], as *P. aeruginosa* is widely recognized as an important opportunistic pathogen in chronic and recurrent canine otitis externa, particularly in cases involving disruption of the normal ear canal microbiota or treatment failure [5].

One possible explanation for the absence of *P. aeruginosa* in the present study is the low frequency of suppurative otitis externa, which was diagnosed in only one dog (1/57; 1.7%). *P. aeruginosa*-associated otitis externa is commonly characterized by suppuration, ulceration, and moderate-to-severe hyperplasia of the ear canal [3, 41], a clinical presentation that was uncommon in the present population. Environmental contamination, including exposure to soil, water, and fomites, and underlying diseases, such as endocrine and immune-mediated disorders, may influence *P. aeruginosa* infections [3, 5, 41]. However, these factors were not evaluated in the present study because environmental samples were not screened for *Pseudomonas* spp. and specific diagnostic investigations for underlying diseases were not performed; therefore, their potential contribution remains uncertain. Importantly, the microbiological methods used in this study were suitable for the recovery of Gram-negative bacteria, including *P. aeruginosa*, and the local MALDI-TOF MS database contained reference spectra for this species [20].

### Occurrence of *Bacillus* spp.

The overall frequency of bacterial isolates belonging to *Bacillus* was 27.5%, comprising *B. licheniformis* (10.0%), *B. cereus* (5.0%), *B. pumilus* (5.0%), *B. megaterium* (2.5%), *B. thermoamylovorans* (2.5%), and *Bacillus* spp. (2.5%). This frequency was higher than those reported in several previous studies. Using Gram staining and biochemical methods for bacterial identification, Lyskova *et al.* [42] reported *Bacillus* spp. in 23.0% and 18.6% of dogs with and without otitis externa, respectively, in the Czech Republic. In Brazil, Casemiro *et al.* [11] reported a frequency of <5% for *Bacillus* spp. among isolates recovered from the canine ototegumentary system, including otitis and pyoderma. Niaraki *et al.* [30] reported that *B. cereus* accounted for 1.3% of bacterial isolates recovered from the ear canals of dogs with otitis externa in Iran. In contrast, using 16S rRNA gene sequencing, Karakaya *et al.* [6] detected *B. cereus* (4.0%) and *B. pumilus* (2.8%) in ear samples from dogs with otitis externa.

The clinical relevance of *Bacillus* spp. recovered in the present study should be interpreted cautiously. Members of this genus are widely distributed in the environment, including soil, dust, and skin surfaces. Karakaya *et al.* [6] suggested that the frequent detection of *Bacillus* spp. in dogs with otitis externa may indicate that environmental bacteria can opportunistically colonize the canine ear canal.

### Fluoroquinolone susceptibility of bacterial isolates

The overall frequency of *in vitro* resistance to both ciprofloxacin and enrofloxacin was 37.5% among the 40 bacterial isolates. Most resistant isolates were recovered from dogs with recurrent otitis externa; however, fluoroquinolone resistance was not significantly associated with recurrence. Reported resistance frequencies for these fluoroquinolones vary considerably among studies. In Serbia, Gram-positive bacteria isolated from the ear canals of dogs with otitis externa showed a relatively low frequency of *in vitro* resistance to enrofloxacin and ciprofloxacin (11% of 40 isolates) [43]. In contrast, among 229 isolates recovered from clinical samples of dogs with otitis externa in China, approximately 25% were resistant to both fluoroquinolones [44]. However, these studies evaluated overall antimicrobial resistance profiles and did not specifically investigate the association between fluoroquinolone resistance and otitis externa recurrence.

Among *S. pseudintermedius* isolates, which represented the predominant bacterial species recovered in the present study, 30% were resistant to both enrofloxacin and ciprofloxacin. This finding is consistent with previous reports from Brazil. Scherer *et al.* [32] reported enrofloxacin resistance in 38.6% of *S. pseudintermedius* isolates recovered from ear samples of dogs with otitis externa. In addition, Brazilian studies evaluating *S. pseudintermedius* isolates recovered from different anatomical sites and clinical conditions, including pyoderma, otitis, and urinary tract infections, have reported ciprofloxacin resistance frequencies ranging from 26% to 49.3% and enrofloxacin resistance of 46.7% [10, 45].

In contrast, some international studies have reported lower resistance frequencies. In Portugal, Saraiva *et al.* [7] reported enrofloxacin resistance in 9% of *S. pseudintermedius* isolates obtained from ear samples of dogs with otitis externa, whereas studies conducted in Serbia, South Africa, and Korea reported resistance to enrofloxacin and/or ciprofloxacin ranging from 13% to 65% [43, 46, 47]. These studies further indicated that fluoroquinolone resistance was more frequently observed among *S. pseudintermedius* isolates carrying the methicillin-resistance gene *mecA* [46, 47].

### Methicillin-resistance and fluoroquinolone-resistant *Staphylococcus* spp.

Although 30% of the *S. pseudintermedius* isolates were resistant to both enrofloxacin and ciprofloxacin, none exhibited phenotypic resistance to oxacillin or cefoxitin, indicating the absence of a methicillin-resistant *S. pseudintermedius* (MRSP) phenotype. This finding contrasts with previous studies in which fluoroquinolone-resistant *S. pseudintermedius* isolates commonly exhibited oxacillin resistance, carried the *mecA* gene, and showed multidrug-resistant profiles [46, 48]. Further molecular characterization is required to determine whether *mecA* is absent from these isolates and to identify the genetic mechanisms underlying their fluoroquinolone resistance.

Other *Staphylococcus* species isolated from the ear samples included *S. hominis* (12.5%), *S. haemolyticus* (2.5%), *S. schleiferi* (2.5%), and *S. sciuri* (2.5%). In addition to the 30% fluoroquinolone resistance observed among *S. pseudintermedius* isolates, all *S. hominis* isolates and the single *S. sciuri* isolate were resistant to fluoroquinolones. Previous studies have reported occasional ciprofloxacin- and enrofloxacin-resistant *S. hominis* and *S. haemolyticus* isolates from dogs with otitis externa, whereas *S. schleiferi* isolates have generally remained susceptible to these antimicrobials [49, 50]. Fluoroquinolone resistance has also been reported among less frequently recovered *S. haemolyticus* and *S. sciuri* isolates from canine skin and mucosal samples obtained at a veterinary hospital, whereas *S. sciuri* isolates recovered from the skin of healthy dogs showed intermediate susceptibility to ciprofloxacin [24, 51].

Despite their low frequency, these findings indicate that *Staphylococcus* species other than *S. pseudintermedius* may also exhibit *in vitro* fluoroquinolone resistance. This finding is clinically relevant because *Staphylococcus* spp. are common members of the normal canine skin and ear microbiota but are also important opportunistic pathogens associated with otitis externa, pyoderma, and systemic infections [24]. In addition to antimicrobial resistance, *S. pseudintermedius* can form biofilms, which may reduce antimicrobial penetration, protect bacteria from host immune defenses, and contribute to persistent or recurrent infections [29]. Biofilm formation was not evaluated in the present study; however, it may pose an additional challenge in treating canine otitis externa. The detection of fluoroquinolone-resistant *Staphylococcus* isolates highlights the importance of continued surveillance of antimicrobial resistance in canine bacterial populations, including less frequently recovered species.

### Fluoroquinolone resistance among other bacterial isolates

Other fluoroquinolone-resistant bacterial isolates identified in the present study included *E. faecium*, *S. liquefaciens*, and *B. licheniformis*. *D. proteolyticus* and *M. radiotolerans* were also classified as resistant according to the interpretive criteria adopted in this study. However, because species-specific breakpoints are unavailable for these organisms, their susceptibility classifications should be interpreted cautiously. All these isolates were recovered from ear samples of dogs with recurrent otitis externa.

*E. faecium* is among the *Enterococcus* species previously reported in canine otitis externa and has been identified as a component of the cerumen microbiota of affected dogs, although at a low relative abundance of 2.8% [6]. Kwon *et al.* [52] reported a high frequency of ciprofloxacin resistance (69.0%) among *E. faecium* isolates recovered from ear samples of dogs with otitis externa. *Serratia* spp. have also been reported in canine otitis externa [11]. Similarly, Li *et al.* [53] identified *Serratia* isolates resistant to ciprofloxacin and enrofloxacin from canine skin and mucosal samples, although resistance occurred in <10% of the isolates.

Published information on fluoroquinolone resistance among *Bacillus* spp. isolated from canine otitis externa is limited. Although one *B. licheniformis* isolate in the present study was resistant to both ciprofloxacin and enrofloxacin, *Bacillus* spp. isolated from canine mucosal and urine samples have generally been reported to be susceptible to fluoroquinolones [54, 55]. To the best of our knowledge, *D. proteolyticus* and *M. radiotolerans* have not previously been reported in ear samples from dogs with otitis externa. Both are primarily environmental microorganisms, and information regarding their occurrence in dogs and antimicrobial susceptibility profiles is scarce.

MALDI-TOF MS enabled the identification of uncommon bacterial species rarely reported in canine otitis externa. However, the clinical significance of these findings remains unclear because information on the occurrence, pathogenic potential, and antimicrobial susceptibility of these microorganisms in dogs is limited. Because some of these species are primarily environmental, their recovery from ear samples may reflect opportunistic involvement or transient colonization. Further studies are required to clarify their role in canine otitis externa.

### Antifungal susceptibility of *M. pachydermatis*

Regarding yeasts, *M. pachydermatis* isolates were susceptible to miconazole but showed resistance to fluconazole (7/16; 43.8%), nystatin (5/16; 31.3%), amphotericin B (2/16; 12.5%), and ketoconazole (1/16; 6.3%). Similar patterns have been reported in two Brazilian studies [12, 13]. Using the disk diffusion method and the same interpretive breakpoints adopted in the present study, Martins *et al.* [12] reported resistance frequencies of 77.8% for fluconazole and 22.2% for both miconazole and ketoconazole among 18 *Malassezia* spp. isolates recovered predominantly from canine ear samples, with a smaller number obtained from cats.

Similarly, Merkel *et al.* [13], using a minimum inhibitory concentration (MIC) method to evaluate the antifungal susceptibility of 15 *M. pachydermatis* isolates recovered from dogs with otitis externa, reported higher MIC values for fluconazole (4–8 µg/mL), consistent with lower *in vitro* activity against *M. pachydermatis*. In comparison, ketoconazole (0.02–0.06 µg/mL) and miconazole (0.05–2 µg/mL) exhibited lower MIC values. Together with the present findings, these Brazilian studies generally indicate lower *in vitro* activity of fluconazole than of miconazole and ketoconazole against *Malassezia* isolates, despite differences in antifungal susceptibility testing methods.

International studies have also reported variable susceptibility profiles of *M. pachydermatis* to the antifungal agents evaluated in the present study. In Chile, Núñez *et al.* [56], using the disk diffusion method, reported susceptibility of isolates recovered from dogs with otitis or seborrheic dermatitis to amphotericin B, fluconazole, and nystatin. Similarly, Yurayart *et al.* [57] in Japan reported susceptibility to ketoconazole and nystatin using disk diffusion and demonstrated good agreement between disk diffusion and MIC results for ketoconazole. In addition, MIC-based studies from Italy [58] and Hungary [59] reported greater *in vitro* activity of ketoconazole against *M. pachydermatis*, as indicated by lower MIC values, whereas miconazole and/or fluconazole exhibited higher MIC values and therefore lower *in vitro* activity. Collectively, disk diffusion and MIC data contribute to the characterization of antifungal susceptibility patterns in *Malassezia* isolates; however, these *in vitro* findings should be interpreted cautiously because they do not necessarily predict clinical response to antifungal therapy [60].

### Antifungal susceptibility and recurrent otitis externa

In the present study, recurrent otitis externa was not significantly associated with recovery of *M. pachydermatis* isolates resistant to at least one antifungal agent (OR = 10.67; p = 0.106), although the numerical difference between the groups warrants further investigation. In addition, three isolates recovered from recurrent otitis externa samples exhibited resistance to fluconazole in combination with resistance to other antifungal agents, including nystatin, amphotericin B, and/or ketoconazole. These findings should be interpreted cautiously because of the small number of *M. pachydermatis* isolates evaluated.

Studies including larger numbers of *M. pachydermatis* isolates from dogs with recurrent and non-recurrent otitis externa are needed to determine whether recurrence is associated with reduced antifungal susceptibility. Treatment histories should also be evaluated to determine whether repeated exposure to topical antifungal agents contributes to the emergence or persistence of isolates with reduced susceptibility. Other potential mechanisms associated with reduced antifungal susceptibility, including biofilm formation by *M. pachydermatis* [61], also warrant investigation.

### Study limitations and future research

Several limitations should be considered when interpreting the findings of this study. The relatively small sample size and inclusion of dogs from a single veterinary hospital may limit the generalizability of the results. In addition, only predominant colonies recovered by aerobic culture were identified, potentially limiting detection of less abundant microorganisms in polymicrobial ear samples. Clinical outcomes, previous treatments, and underlying diseases associated with recurrent otitis externa were not evaluated, limiting assessment of factors potentially related to antimicrobial resistance. Furthermore, species-specific susceptibility breakpoints were unavailable for some uncommon bacterial isolates, requiring cautious interpretation of their resistance classifications. Finally, although antimicrobial susceptibility testing provided relevant information on *in vitro* resistance patterns, these findings do not necessarily predict clinical treatment response.

Future studies should include larger and more geographically diverse canine populations, longitudinal follow-up, detailed treatment histories, investigation of underlying diseases, and molecular characterization of resistance mechanisms. Culture-independent approaches may also provide a more comprehensive assessment of the microbial communities associated with recurrent and non-recurrent otitis externa.

### Antimicrobial stewardship implications

The *in vitro* antimicrobial resistance patterns observed in this study should also be considered within the broader context of antimicrobial stewardship in veterinary medicine in Brazil. Topical otic formulations combining antibiotics, antifungals, and corticosteroids are widely available in the Brazilian market, and inappropriate or repeated empirical use may increase antimicrobial selection pressure within the ear canal microbiota [7, 12]. Accordingly, microbiological evaluation and antimicrobial susceptibility testing are particularly relevant in recurrent infections and cases that respond poorly to empirical therapy.

The Brazilian Ministry of Agriculture, Livestock and Food Supply published the *Guide for the Rational Use of Antimicrobials in Dogs and Cats* in 2022 [62], emphasizing the risks associated with inappropriate antimicrobial use in companion animals. The resistance patterns observed in the present study reinforce the importance of rational antimicrobial use, appropriate diagnostic investigation, and continued surveillance of antimicrobial susceptibility among microorganisms recovered from dogs with otitis externa.

## CONCLUSION

This study characterized the clinical, cytological, microbiological, and antimicrobial susceptibility profiles of dogs with recurrent and non-recurrent otitis externa in Brazil. Recurrent otitis externa was not significantly associated with greater otoscopic or cytological severity, positive bacterial or yeast cultures, or the presence of fluoroquinolone-resistant bacterial isolates. Bacterial proliferation was the most frequent cytological finding and showed the strongest association with the distribution of observations in the log-linear Poisson model (RR = 18.0; p < 0.001). *Staphylococcus* was the predominant bacterial genus, with *S. pseudintermedius* being the most frequently isolated species. Resistance to both ciprofloxacin and enrofloxacin was detected in 37.5% of bacterial isolates, including 30% of *S. pseudintermedius* isolates; however, none of the fluoroquinolone-resistant *S. pseudintermedius* isolates exhibited a methicillin-resistant phenotype. Among *M. pachydermatis* isolates, resistance was most frequent to fluconazole and nystatin, whereas all isolates were susceptible to miconazole. Although recurrent otitis externa was not significantly associated with antifungal resistance, the higher proportion of *M. pachydermatis* isolates resistant to at least one antifungal agent among recurrent cases warrants further investigation.

The detection of fluoroquinolone-resistant bacteria and *M. pachydermatis* isolates with reduced susceptibility to commonly used antifungal agents emphasizes the importance of integrating cytological examination, microbial culture, and antimicrobial susceptibility testing into the diagnostic evaluation of canine otitis externa, particularly in recurrent or poorly responsive cases. The findings also support prudent use of topical antimicrobial formulations and continued antimicrobial resistance surveillance to guide therapeutic decisions and antimicrobial stewardship in companion-animal practice.

A major strength of this study was the integrated assessment of clinical and otoscopic findings, cytology, microbiological culture, MALDI-TOF MS-based identification, and *in vitro* antimicrobial susceptibility within the same population of dogs. Direct comparison of recurrent and non-recurrent cases also provided locally relevant baseline information on microbial distribution and susceptibility patterns in canine otitis externa in Brazil.

The findings indicate that recurrent and non-recurrent canine otitis externa share broadly similar clinical and microbiological characteristics, while clinically relevant antimicrobial resistance occurs among bacterial and yeast isolates recovered from affected ears. These results provide baseline data for surveillance and reinforce the need for evidence-based antimicrobial selection rather than repeated empirical treatment. Larger multicenter and longitudinal studies incorporating treatment history, underlying diseases, molecular characterization of resistance mechanisms, biofilm assessment, and culture-independent microbial profiling are warranted to clarify factors contributing to recurrence and antimicrobial resistance in canine otitis externa.

## DATA AVAILABILITY

The supplementary data can be available from the corresponding author upon a request.

## GENERATIVE AI DECLARATION

The authors declare that generative artificial intelligence (AI) tools were used solely to improve language, grammar, and readability during manuscript preparation. All scientific content, data analysis, interpretation of results, and conclusions were developed and verified by the authors. The authors take full responsibility for the accuracy, integrity, and originality of the work presented, and no AI tool was listed as an author.

## AUTHORS’ CONTRIBUTIONS

LGO, RLMS, and DCALD: Conceived and designed the study. LGO and CBP: Performed sample and data collection. LGO, CBP, and FSM: Conducted laboratory investigations and processed the samples. FSM and RLMS: Provided laboratory assistance and technical support. LGO, CDC, FEC, and DCALD: Performed data curation, formal analysis, and wrote the original draft of the manuscript. FEC and DCALD: Performed data validation and statistical analysis. DCALD: Supervised the research. All authors have read and approved the final version of the manuscript.
